# Tetrazolium Compounds: Synthesis and Applications in Medicine

**DOI:** 10.3390/molecules20045528

**Published:** 2015-03-27

**Authors:** Cheng-Xi Wei, Ming Bian, Guo-Hua Gong

**Affiliations:** 1 Medicinal Chemistry and Pharmacology Institute, Inner Mongolia University for the Nationalities, Tongliao 028000, Inner Mongolia, China; E-Mails: weichengxi1224@163.com (C.-X.W.); xiaopang1224@126.com (M.B.); 2 Affiliated Hospital of Inner Mongolia University for Nationalities, Tongliao 028000, Inner Mongolia, China

**Keywords:** tetrazole, sythesis, antibacterial, anti-inflammatory, anticancer, anticonvulsant

## Abstract

Tetrazoles represent a class of five-membered heterocyclic compounds with polynitrogen electron-rich planar structural features. This special structure makes tetrazole derivatives useful drugs, explosives, and other functional materials with a wide range of applications in many fields of medicine, agriculture, material science, *etc.* Based on our research works on azoles and other references in recent years, this review covers reported work on the synthesis and biological activities of tetrazole derivatives.

## 1. Introduction

**Figure 1:**
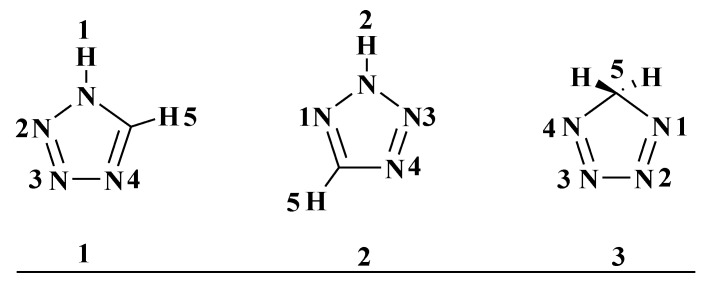
Structures of the regioisomeric tetrazole rings.

Tetrazole is a heterocyclic compound containing a carbon atom and four nitrogen atoms in a five-membered ring. Theoretically there are three precursor tetrazole isomers: *i.e.*, 1*H*-tetrazole (**1**), 2*H*-tetrazolium (**2**) and 5*H*-tetrazole (**3**) (Figure 1). Substituted tetrazoles exist as a nearly 1:1 ratio of 1*H*- and 2*H*-tautomeric forms. Previous studies had shown that the two positional isomers **1** and **2** may be differentiated on the nuclear magnetic resonance (NMR) timescale [1].

Like other azole compounds, due to the relatively late start of the synthesis and study of tetrazole compounds, they did not attracted much attention in the beginning. Since 1885, when Bladin first synthesized tetrazole derivatives (2-cyanophoric-5-phenyltetrazole) to 1950, only some 300 kinds of derivatives were reported [2]. Since the 1950s, when tetrazole compounds became widely used in agriculture, biochemistry, medicine, pharmacology, explosives and other aspects, research began to develop rapidly [3,4]. The tetrazolyl functional group which was often considered as a carboxylic acid replacement in drugs, not only because the pKa is close, but it also has approximately the same planar delocalized system space requirements, and it provided a maximum nitrogen content of any heterocyclic compound [5]. The planar ring skeleton structure and the nitrogen-rich multi-electron conjugated system confer tetrazole derivatives with both donor and acceptor electronic properties. Tetrazole and its derivatives have this attracted the interest of scientists because of their unique structures and their potential applications as antihypertensive, anti-allergic, antibiotic and anticonvulsant agents [6,7,8,9]. In the present review, emphasis was focused on the diverse pharmacological properties associated with substituted tetrazoles in the past few years and a conclusive discussion on structure-activity relationship (SAR) of these compounds is provided.

## 2. Preparation of Tetrazole Derivatives

**Scheme 1:**
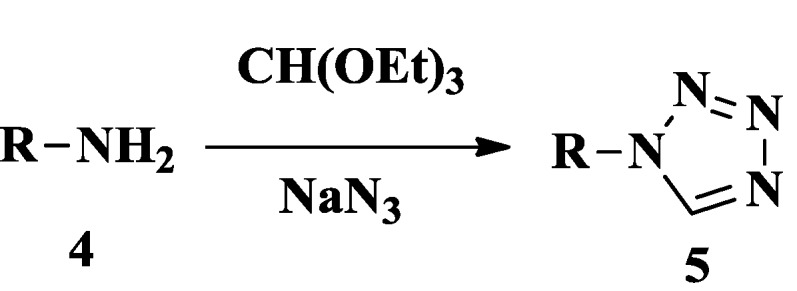
Synthesized route 1 for tetrazoles.

**Scheme 2:**
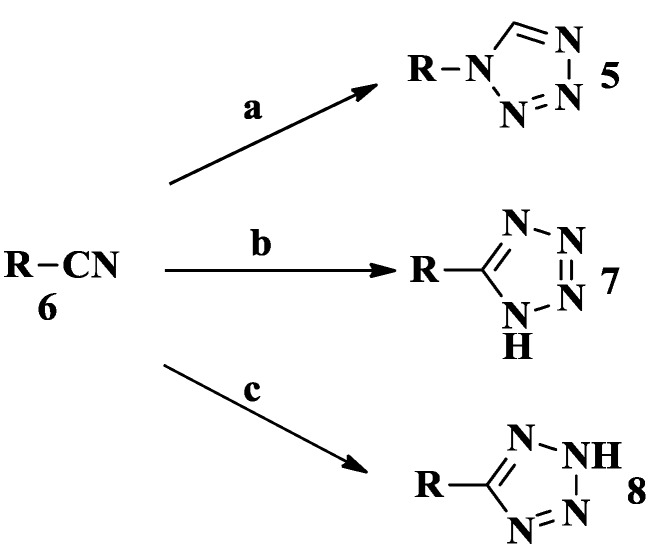
Synthesized route 2 for tetrazoles.

**Scheme 3:**
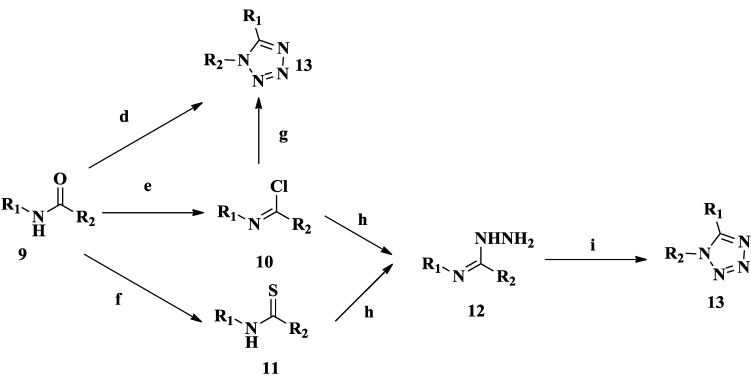
Synthesized route 3 for tetrazoles.

Several methods for the synthesis of tetrazoles are widely reported in the literature. The main synthetic routes to tetrazoles are outlined in Scheme 1, Scheme 2 and Scheme 3. Tetrazoles could be synthesized by the reaction of substituted amines **4** with triethyl orthoformate and sodium azide in dimethyl sulfoxide (DMSO) [10,11] (Scheme 1). A new method to convert substituted amines into tetrazoles involves preparing functionalized superparamagnetic Fe_3_O_4_·SiO_2_ possessing high saturation magnetization [12].

The [3+2] cycloaddition between hydrazoic acid and cyanide derivatives is well known as an efficient route (Scheme 2). We can thus get substituted tetrazoles **5** from isocyanides **6**. The reaction of **6** and azidotrimethylsilane (1.5 equiv.) was conducted in MeOH (0.5 M) in the presence of a catalytic amount of HCl (2 mol %, 1.0 M in Et_2_O solution) at 60 °C [13,14]. One of the main procedures frequently used for the preparation of 5-substituted tetrazoles **7** involves heating a suspension of **6**, sodium azide, ammonium chloride and lithium chloride (1.20 g, 28 mmol) in anhydrous dimethylformamide (DMF) under stirring at 110 °C [15,16,17]. Compound **7** was also obtained in a sealed pressure vessel reaction where NaN_3_ dissolved in H_2_O, compound **6**, ammonium chloride, ammonium fluoride and propane-1,2-diol (DPOL) were stirred and heated for 48 h [18]. Aryloxy tetrazoles were commonly prepared by the reaction of phenols with cyanogen bromide in the first step. Then sodium azide was added into refluxing acetone-water [19]. The most convenient route to 5-substituted-1*H*-tetrazoles **8** is the reaction of azide ion with nitriles [20]. The literature is full of examples of this conversion method. They can be divided into three main categories using tin or silicon azides [21], acidic media [22], and strong Lewis acids [23,24]. In addition, all of the known methods use organic solvents, in particular, dipolar aprotic solvents such as DMF [25]. A new method for the synthesis of 5-substituted-1*H*-tetrazoles method was reported where compound **6**, water, sodium azide and zinc chloride were refluxed in a hood [26,27,28].

*Reagents and Conditions*: a: MeOH, HCl, 60 °C; b: (1) NaN_3_, NH_4_Cl, LiCl, DMF, 110 °C; (2) NaN_3_, NH_4_Cl, NH_4_F, DPOL, 48 h; c: N_3_^−^, CN^−^.

If one wants to prepare 1,5-disubstituted-tetrazoles from the corresponding amide, there are many routes (Scheme 3). First, one can prepare an oxyphosphonium salt under Mitsunobu conditions from the corresponding amide, followed with reaction with trimethylsilyl azide leading to the desired 1,5-disubstituted-tetrazoles. This method proves to be especially useful for applications when an N-protected tetrazole is required. Preservation of chirality in the synthesis of a tetrazole analogue of an amino acid (phenylalanine) was demonstrated [29]. In addition to the above direct approach, we can also through multiple steps to generate 1,5-disubstituted tetrazoles. Usually compounds **9** can be converted to compounds **10** with phosphorus oxychloride (POCl_3_) and thionylchloride (SOCl_2_) [30]. Compounds **11** can also be prepared in suitable yields by reaction of compounds **9** with Lawesson’s reagent or phosphorus pentoxide [31]. 1,5-Disubstituted tetrazoles can also be synthesized by reacting compounds **10** with triethyl orthoformate and sodium azide [32]. Compounds **12** can be synthesized by the reaction of compounds **10** and compounds **11** with hydrazine hydrate [33,34]. 1,5-Disubstituted tetrazoles can finally be synthesized by reacting **12** with CH_3_COOH and aqueous NaNO_2_ under ice-bath conditions, making sure that the reaction temperature is below 5 °C [35,36].

*Reagents and Conditions*: d: oxyphosphonium salt, C_3_H_9_N_3_Si; e: POCl_3_, SOCl_2_; f: Lawesson’s reagent/P_2_O_5_; g: HC(OC_2_H_5_)_3_, NaN_3_; h: N_2_H_4_·H_2_O; i: CH_3_COOH, NaNO_2_, below 5 °C.

**Scheme 4:**
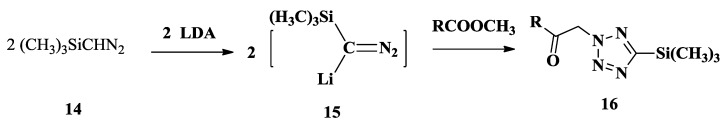
The most efficient route for synthesis of 2,5-disubstituted-tetrazoles.

There are some ways to get 2,5-disubstituted-tetrazoles. One of the most efficient routes (Scheme 4) uses lithium trimethylsilyldiazomethane (**15**) prepared from TMSCHN_2_ and lithium diisopropylamide (LDA), which reacts smoothly with the methyl esters of carboxylic acids at 0 °C to give compounds **16** in good yields [37].

**Scheme 5:**
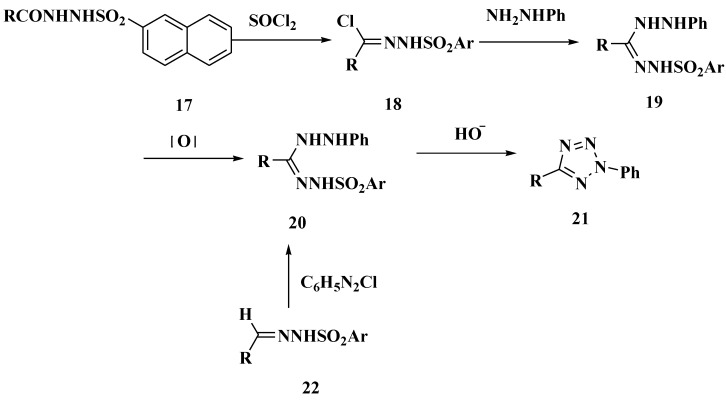
Synthesized routes for compounds 18–22.

When the hydrazides **17** were treated with thionyl chloride, the corresponding hydrazidoyl compounds **18** were obtained (Scheme 5). Next the reaction of compounds **18** with phenylhdyrazine was examined to prepare the hydrazidines **19** which can be oxidized to formazan derivatives **20**. 2,5-Disubstituted tetrazoles **21** can be synthesized by reacting compounds **20** with potassium carbonate [38]. Compounds **20** also can be synthesized from compounds **22** [39].

## 3. Biological Activity of Tetrazolium Derivatives

Compounds derived from tetrazolium have received particular attention due to their pharmacological properties. Numerous studies have been published on the antibacterial and antifungal properties of these derivatives. Furthermore, these compounds also present anti-inflammatory, analgesic, anticancer, anticonvulsant, antihypertensive, hypoglycemic, antiparasitic, and antiviral activities*.* The aforementioned properties and the possibility to attach several structurally distinct substituents to the heterocycle ring to modify either the biological or physico-chemical properties of these compounds have prompted the use of this heterocycle as a template in many research programs aimed at the development of new bioactive compounds.

### 3.1. Antibacterial and Antifungal Activity

**Figure 2:**
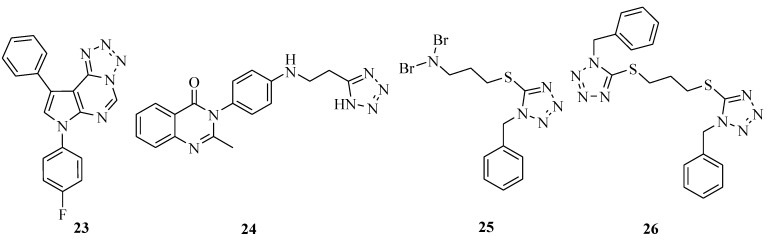
Structures of compounds **23**–**26**.

7,9-Disubstituted-7*H*-tetrazolo[1,5-*c*]pyrrolo[3,2-*e*]pyrimidines (Figure 2) were synthesized and evaluated for their antibacterial activity. Compound **23** exhibited better activity than ampicillin against all the tested cultures, except *S. aureous* [40]. A novel series of tetrazole compounds were reported to possess antimicrobial activity *in vitro* by the disc diffusion method measuring zones of inhibition. The results of the study showed that the synthesized compound 2-methyl-3-{4-[2-(1*H*-tetrazol-5-yl-ethylamino]phenyl}-3*H*-quinazolin-4-one (**24**) displayed fairly good antimicrobial activity against the test organisms, although the activity was less than that of the reference drugs (ciprofloxacin and fluconazole, respectively) [41]. It was reported that a series of new 5-thio-substituted tetrazole derivatives were synthesized and antimicrobial screening showed that all the synthesized compounds showed moderate activity against the tested organisms. Among the newly synthesized compounds, **25** and **26** showed the most effective antibacterial and antifungal activities. The study suggested that further work with similar types of analogues was clearly warranted [42].

**Figure 3:**
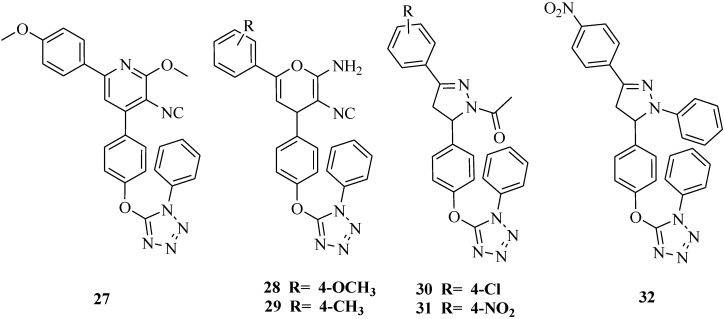
Structures of compounds **27**–**32**.

A variety of heterocyclic tetrazole derivatives were synthesized (Figure 3). Among the synthesized compounds, compounds **27**, **28**, **29**, **30**, **31** and **32** exhibited antimicrobial activities with minimal inhibitory concentration (MIC) values ranging from 23.40 to 46.87 μg/L. Molecular modeling results were in accordance with the *in vitro* antimicrobial screening. The SAR research results showed that pyran derivatives were more active than pyridine derivatives and the activity order for the R substituent was: *4*-OMe> *4*-Me > *3*-OH > H > *4*-Cl > *4*-NO_2_ [43].

**Figure 4:**
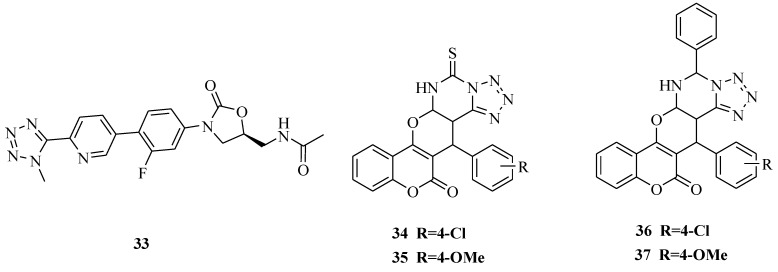
Structures of compounds **33**–**37**.

A new series of oxazolidinone derivatives were synthesized (Figure 4) and evaluated their substituted effects on *in vitro* and *in vivo* antibacterial activities activity against clinically relevant resistant gram-positive organisms, *M. catarrhlis* and *H. influenzae* with a long half-life.

Diverse substituted heteroaromatic ring forms were tolerated on the pyridine and the presence or orientation of the methyl group in the heteroaromatic ring affected the antibacterial activity. Among the new compounds, **33** was the most effective compound against a variety of clinically relevant resistant Gram-positive organisms and *M. catarrhlis* and *H. influenzae*, and also showed the higher *in vivo* efficacy with longer half-life than linezolid [44]. The synthesis of a series of novel pyranochromene-containing fused tetrazole derivatives was reported. All of the newly synthesized compounds were screened for their antibacterial activities against the Gram-positive bacteria *Bacillus subtilis* and *Staphylococcus aureus* and Gram-negative bacteria (*Pseudomonas aeuroginosa* and *Escherichia coli*)*.* The antifungal activity of the compounds was assayed against *Candia albicans* and *Aspergillus niger*. The MIC values of the assayed compounds were determined using the microdilution susceptibility method. Ciprofloxacin was used as a reference antibacterial agent. Fluconazole was used as a reference antifungal agent. The study showed that compounds **34**, **35**, **36** and **37** showed significant antibacterial and antifungal activities compared with other tested samples [45].

**Figure 5:**
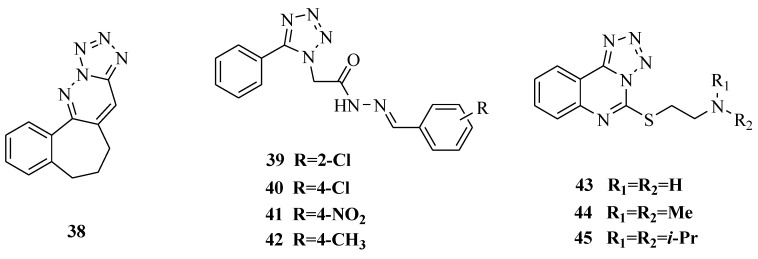
Structures of compounds **38**–**45**.

The synthesis of new triazolo/tetrazolo-pyridazine[6,7]benzocycloheptenes (Figure 5) and their antimicrobial activity was reported. All the compounds were screened for the antibacterial activity in the appropriate concentration. The compound 2-methyl-6,7-dihydro-5*H* benzo[6,7]cyclohepta[1,2,3,4] tetrazolo[1,5-*b*] pyridazine (**38**) showed the maximum zone of inhibition (20 mm) against *E.coli* which was a little smaller than that of doxycyclin (30 mm) [46]. A series of tetrazole Schiff bases were prepared and it was demonstrated that these compounds possessed good antibacterial and antifungal activities when tested by the cup plate method. The most promising compounds with good antibacterial and antifungal activity were **39**, **40** and **41**, and the best ones for antifungal activity were **39**, **40** and **42** [47].

**Figure 6:**
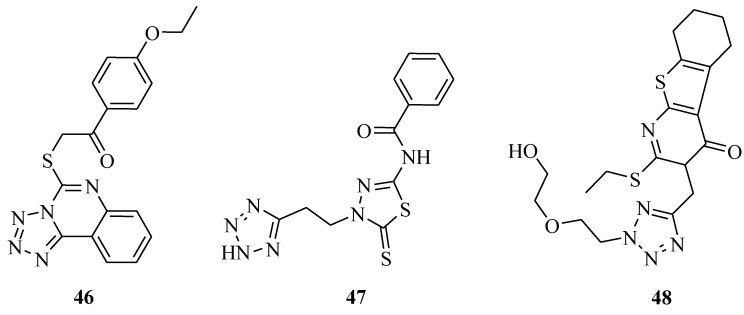
Structures of compounds **46**–**48**.

It was reported that a series of tetrazolo[1,5-*c*]quinazoline-5-thione derivatives (Figure 5 and Figure 6) were synthesized and screened for antibacterial and antifungal activities at the concentration of 100 μg*.* The results revealed that the most active substances were **43**, **44**, **45**, and **46**. The SAR revealed that shortening of the dialkyl amino fragment of substances **43**–**45** leads to a moderate decrease in antimicrobial activity against *Enterococcus faecalis*, and otherwise leads to an increase in activity against *Staphylococcus aureus* and *Escherichia coli*, and antifungal activity against *Candida albicans*. Introduction of the 4-methoxyphenyl group at the 5 position of tetrazolo[1,5-*c*]quinazoline-5-thione (**46**) resulted in the best antimicrobial agent among all the investigated substances. It even moderately inhibited the growth of *Pseudomonas aeruginosa* and *Klebsiella pneumoniae* [48]. Other research results showed that *N*-(4-(2-(2*H*-tetrazol-5-yl)ethyl)-5-thioxo-4,5-dihydro-1,3,4-thiadiazol-2-yl)-benzamide (**47**) exhibited antimicrobial activity against *Bacillus subtilis* with a MIC value of 100 μg/mL compared with penicillin (31 μg/mL), against *Pseudomonas aeruginosa* with a MIC value of 125 μg/mL compared with penicillin (46 μg/mL), and against *Streptomyces species* with a MIC value of 125 μg/mL compared with penicillin (33 μg/mL) [49]. A series of substituted 5,6,7,8-tetrahydro-3*H*-benzo[4,5]thieno[2,3-*d*]pyrimidine-4-one derivatives were synthesized and evaluated for antimicrobial activity against *Escherichia coli*, *Bacillus subtilis*, *Staphylococcus aureus*, *Aspergillus niger* and *Candida albicans*. The substituted thienopyrimidine derivative (**48**) was the most highly active compound [50].

**Figure 7:**
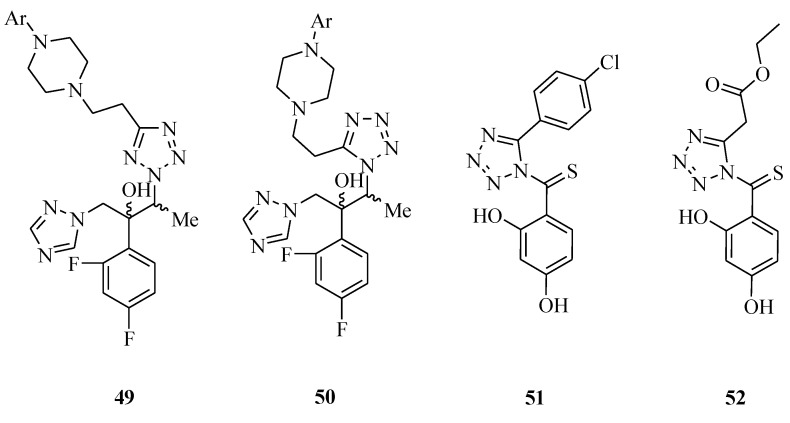
Structures of compounds **49**–**52**.

A variety of triazole derivatives with a 5-substituted tetrazole structure were prepared (Figure 7) and evaluated for their antifungal activity against the *Aspergillus* spp, *Cryptococcus neoformans*, and *Candida* spp. *in vitro*. Some of these compounds possessed good antifungal activity against the different fungal cultures such as *Candida* species, *C. neoformans* and *Aspergillus* species. The location of the methyl group at the C-3 of compound **49** and **50** has been demonstrated to be a key structural element for antifungal potency [51]. It was reported that various 1-(2,4-dihydroxythiobenzoyl)imidazoles, -imidazolines and -tetrazoles were synthesized and evaluated for antibacterial properties. The MIC values against the *Candida albicans* ATCC 10231 strain, azole-resistant clinical isolates of *C. albicans* and non-*Candida* species were determined. Tetrazole derivatives **51** and **52** were the most active against *C. albicans*. All compounds showed higher activity than reference drugs itraconazole and fluconazole [52].

**Figure 8:**
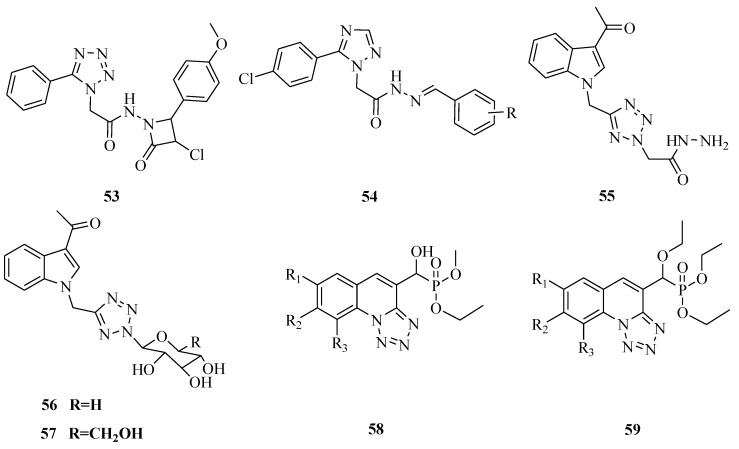
Structures of compounds **53**–**59**.

Novel tetrazole derivatives containing azatidinone (Figure 8) were designed, synthesized and evaluated for antitubercular activity. The highest activity registered for compounds was **53** which should be regarded as a new hit for further development as a novel class of anti-*Mycobacterium tuberculosis* agents [53]. A series of tetrazole -earing acylhydrazone derivatives **54** were synthesized. Activity evaluation of some of these compounds showed anti-fungicidal activity [54]. A series of new 1-[(tetrazol-5-yl)methyl]indole derivatives were also synthesized. Compound **55** displayed strong activity against *Escherichia coli*, *Bacillus subtilis*, *Streptococcus lactis* and *Pseudomonas sp.*, compared with ciprofloxacin and exhibited high activities against the tested fungi compared with fusidic acid, while compounds **56** and **57** showed strong activity against the Gram negative bacteria *Escherichia coli* and *Pseudomonas aeruginosa* [55]. New *a*-hydroxyphosphonate **58** and *a*-acetoxyphosphonate derivatives **59** were synthesized and their antimicrobial activities evaluated. All compounds demonstrated potent inhibition against all the strains tested [56].

### 3.2. Anti-Inflammatory and Analgesic Activity

**Figure 9:**
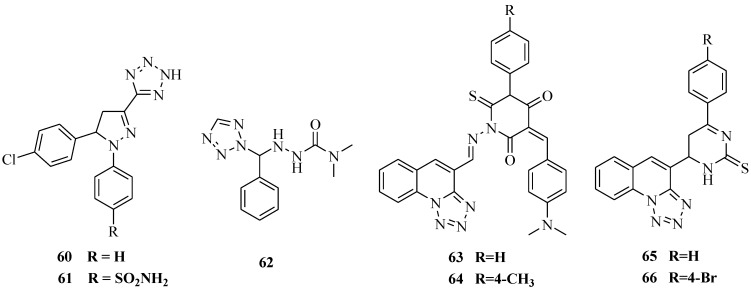
Structures of compounds **60**–**66**.

It was reported that a series of 4,5-dihydro-1,5-diaryl-1*H*-pyrazole-3-substituted-heteroazoles were designed and synthesized (Figure 9). All the compounds were screened for anti-inflammatory activity using the carrageenan-induced rat paw edema method. Diclofenac sodium was used as a standard drug for comparison. Compound **61** which is a tetrazole derivative was found to be the most potent anti-inflammatory compound in the present series as compared to the reference. Even the des-sulfonamide 1,2,4-oxadiazole derivatives **60**, **61** showed good *in vivo* anti-inflammatory potency, meriting further attention in order to develop new leads in this series [57]. A new series of novel substituted tetrazole derivatives were prepared by reacting tetrazole with different types of carbazone derivatives and various substituted type of benzaldehyde were thus synthesized. All synthesized compounds were screened for anti-inflammatory activity in rats by the carrageenan-induced paw oedema method at a dose of 50 mg/kg body weight. All compounds showed moderate enhancement of the activity. Among the tested compound, compound **62** exhibited good potential anti-inflammatory activity when compared to the standard phenylbutazone (PBZ) at 5 mg/kg/po [58]. Three series of tetrazolo[1,5-*a*]quinoline derivatives were synthesized. All the test compounds significantly inhibited granuloma formation. It could be safely concluded that the median effective dose (ED_50_) values of compounds **63**, **64**, **65** and **66** which range from 8.50 to 9.84 μmol have anti-inflammatory activity comparable to that of indomethacin (ED_50_ value = 9.28 μmol) [59].

**Figure 10:**
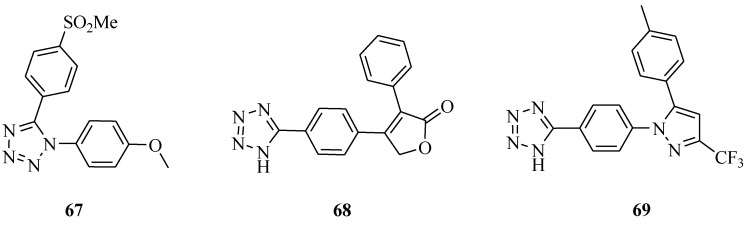
Structures of compounds **67**–**69**.

It was reported that a novel series of 1,5-diaryl-substituted tetrazole derivatives (Figure 10) were synthesized and evaluated for their anti-inflammatory activity. All tetrazoles showed half maximal (50%) inhibitory concentration (IC_50_) values ranging from 0.42 to 8.1 mM for COX-1 and 2.0 to 200 μM for COX-2. The most potent compound **67** (IC_50_ (COX-2) = 2.0 μM) was further used in molecular modeling docking studies [60]. New water-soluble, parenteral COX-2 inhibitor rofecoxib (compound **68**) and celecoxib (compound **69**) analogues were designed, synthesized and evaluated as selective cyclooxygenase-2 (COX-2) inhibitors with *in vivo* anti-inflammatory activity. The rofecoxib and celecoxib analogues **68** and **69** exhibited a high *in vitro* selectivity (**68**, COX-1 IC_50_ = 3.8 nM; COX-2 IC_50_ = 1.8 nM; SI = 2.11; **69**, COX-1 IC_50_ = 4.1 nM; COX-2 IC_50_ = 1.9 nM; SI = 2.16) relative to the reference drug celecoxib (COX-1 IC_50_ = 3.7 nM; COX-2 IC_50_ = 2.2 nM; SI = 1.68) and also showed good anti-inflammatory activity in a carrageenan-induced rat paw edema assay and high aqueous solubility at pH values higher than 7. Also **68** and **69** caused no significant damage to the gastric mucosa [61].

**Figure 11:**
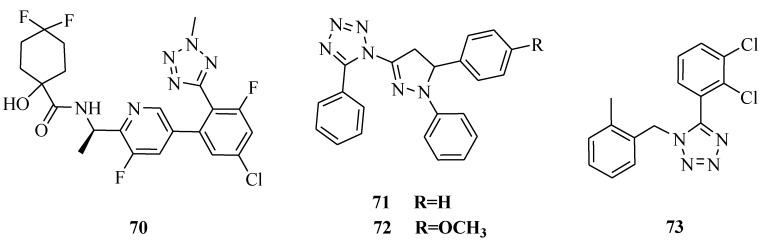
Structures of compounds **70**–**73**.

It was reported that a series of 5- and 6-membered carbocyclic and heterocyclic α-hydroxy amide-derived bradykinin B1 antagonists bearing an *N*-2 methyltetrazole as an oxadiazole replacement were prepared (Figure 11) and evaluated. A number of compounds with excellent B1 binding affinity, good pharmacokinetic properties, and desirable P-gp transport properties were discovered. In particular, compound **70** exhibited excellent binding affinity (hKi = 0.41 nM), P-gp transport, and dog pharmacokinetic profiles (F = 91%) [62]. Various 1-[5-(substituted phenyl)-1-phenyl-4,5-dihydro-1*H*-pyrazol-3-yl]-5-phenyl-1*H*-tetrazoles were also synthesized. All the compounds exhibited weak to potent anti-inflammatory activity. The compounds **71** and **72** possessed potent anti-inflammatory activity in comparison with ibuprofen [63]. A series of selective P2X_7_ receptor antagonists for chronic inflammation and pain ATP, acting on P2X_7_ receptors was reported. Although highly potent compounds such as compound **73** were discovered, the poor physiochemical properties of these molecules led to an investigation of heterocyclic attachments that might confer improved properties in this regard [64].

**Figure 12:**
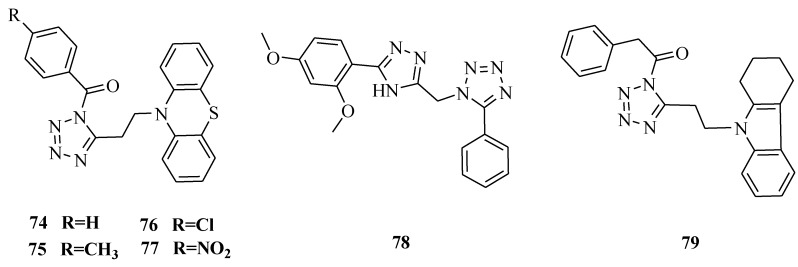
Structures of compounds **74**–**79**.

Synthesis of novel 5-[β-(phenothiazinyl-10-yl)ethyl]-1-(acyl)-1,2,3,4-tetrazoles was reported (Figure 12). The synthesized compounds were screened for analgesic and anti-inflammatory activity. Out of the 12 compounds synthesized, compounds **74** and **75** showed promising analgesic activity, while compounds **76** and **77** showed promising anti-inflammatory activity [65].

1,2,4-Triazole derivatives containing pyrazole, tetrazole, isoxazole and pyrimidine rings were synthesized and evaluated for analgesic activity *in vivo*. The hot plate and acetic acid induced writhing methods have been described to study analgesic activity. Compound **78** exhibited comparative analgesic properties (up to 60% protection) to the standard drug ibuprofen (66%). The analgesic activity measured by the central nervous system analgesic pentazocine (5 mg/kg) significantly increased the hot plate latency producing a highest % MPE at 69.02. Compound **78** had 60.62% MPE [66]. Twelve different derivatives of substituted-{5-[2-(1,2,3,4-tetrahydrocarbazol-9-yl)ethyl]tetrazol-1-yl}alkanones were synthesized. The compounds were screened for antinociceptive activity by the acetic acid induced writhing method and hot plate method. Compound **79** was found to be the most active compound of the series [67].

### 3.3. Anticancer Activity

**Figure 13:**
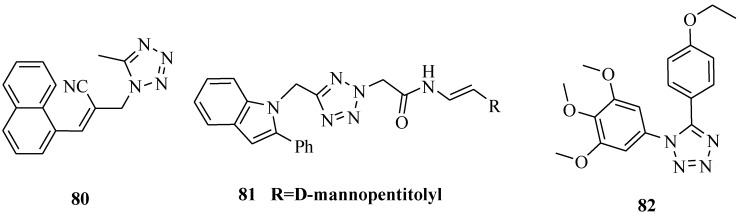
Structures of compounds **80**–**82**.

It was reported that a series of new tetrazole derivatives were synthesized from Baylis-Hillman allyl amines in a clean, efficient and straightforward manner (Figure 13). Compound **80** was found particularly more active against liver carcinoma (Hep G2) and lung adenocarcinoma (A 549) cancer cell lines, while compound **80** also displayed significant activity against prostate (DU 145) cancer cell line. The hit compound **80** could bind to DNA and form a stable complex and may act as a potential genotoxic agent for cancer therapy [68].

New (tetrazol-5-yl)methylindole derivatives were synthesized from 2-phenylindole. The synthesized compounds were studied for their anticancer activity against the human liver carcinoma cell line HepG2 and the results showed that compound **81** was highly active among the series of tested compounds and it affected the cell viability in a dose dependent manner with an IC_50_ value of 4.2 μM [69]. Two series of 1,5-diaryl-substituted-1,2,3,4-tetrazoles were concisely synthesized. Several of the synthesized compounds also had effective activity in inhibiting the growth of multidrug resistant cells overexpressed P-glycoprotein. Compound **82** was a strong inhibitor of tubulin polymerization (IC_50_ = 1.1 μM) and strongly inhibited the binding of colchicine to tubulin (78% inhibition). The results demonstrated that **82** is a promising new tubulin binding agent worthy of further evaluation as a potential chemotherapeutic agent [70].

**Figure 14:**
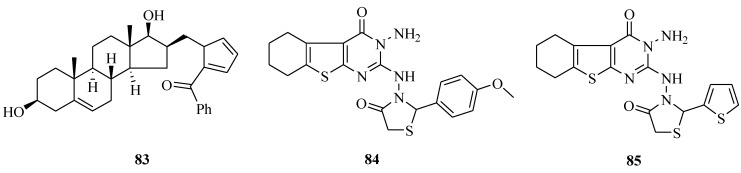
Structures of compounds **83**–**85**.

It was reported that a synthesis of several D-ring-substituted steroidal tetrazoles were prepared by means of 1,3-dipolar cycloadditions (Figure 14). The novel synthesized compounds were screened for their activities against a panel of three human gynecological cancer cell lines (HeLa, MCF7 and A2780). The study showed that of all these compounds only **83** had a moderate effect [71]. Some new spirothieno[2,3-*d*]pyrimidine, imidazolidine, substituted pyrimidinyl and substituted thiazolidine thieno[2,3-*d*]prymidine derivatives were synthesized. The combination of the potent anticancer activity of compounds **84** and **85** with their less toxicity and the ease of synthesis makes them the promising lead compounds for cancer chemotherapy [72].

**Figure 15:**
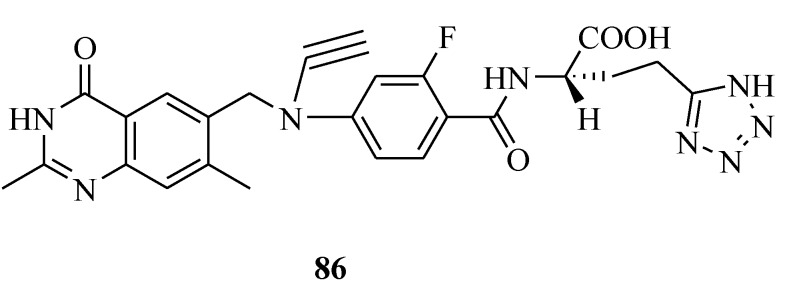
Structure of compound **86**.

Jackman *et al*., reported a drug ZD9331 (**86** in Figure 15) which exhibits potent growth inhibitory and cytotoxic activity. The results suggested that ZD9331 would have a different, or at least an incompletely overlapping spectrum of antitumor activity and toxicity profile, compared with tomudex and possibly other TS inhibitors currently being studied clinically [73].

A series of new 1,2-substituted tetrazole derivatives were synthesized and evaluated on MCF-7 (ER positive), MDA-MB-231 and ZR-75 (ER negative) breast cancer cell lines. Compounds **87**, **88** and **89** showed higher inhibitory effects on MCF-7 cells, whereas compound **90** exhibited a higher inhibition on MDA-MB-231 cells and ZR-75 cells at a concentration of 10^−5^ M. To know if the compounds are selectively targeting specific cancers, they were screened against the HepG2 cell line. Only 1%–10% inhibition was found at 10^−5^ M concentration [74]. A series of 1,5-disubstituted tetrazole-tethered combretastatin analogues were developed and evaluated for their antitubulin and antiproliferative activity. Compounds **91** and **92**, having hydrogen-bonding donor groups at the *ortho-* and *meta*-positions on the 4-methoxyphenyl B ring, are strong inhibitors of tubulin polymerization and antiproliferative agents having IC_50_ values in micromolar concentrations [75].

**Figure 16:**
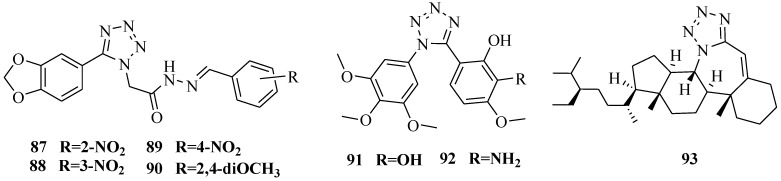
Structures of compounds **87**–**93**.

A novel synthetic molecule 7a-aza-B-homostigmast-5-eno[7*a*,7-*d*] tetrazole was reported (Figure 16). The compound **93** was tested against two human cancer cell lines: CT116 and HepG2 and one non-cancerous HFL1 (human lung fibroblast) cell line. The IC_50_ values for all compounds were compared to doxorubicin. The results indicated that the compound **93** inhibited the various cancer cell lines in a dose-dependent manner and effectively inhibited cancer cell growth [76].

### 3.4. Anticonvulsant Activity

**Figure 17:**
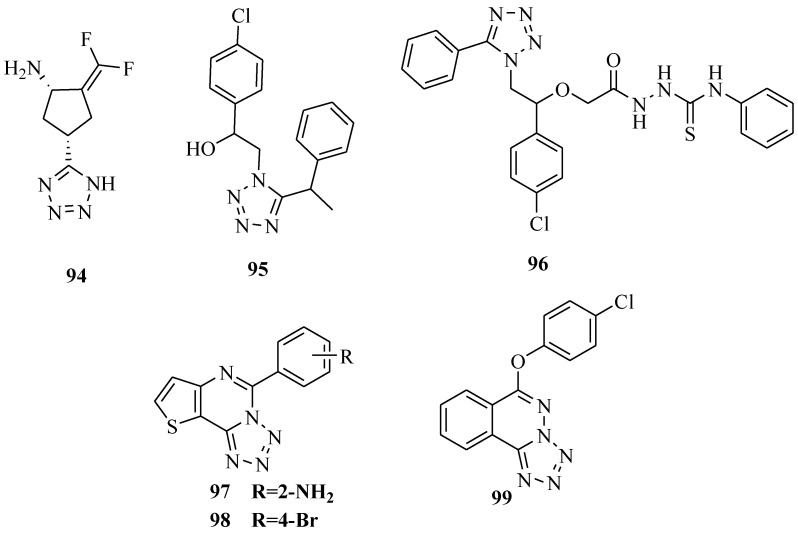
Structures of compounds **94**–**99**.

A potent inactivator of GABA-AT was synthesized (Figure 17), which was shown to be a time- and concentration-dependent GABA-AT inhibitor. The study found low brain levels of the inhibitory neurotransmitter γ-aminobutyric acid (GABA) can lead to seizure. Inhibition of GABA aminotransferase can increase the concentration of GABA and can terminate the convulsions. The K_inact_/KI value for compound **94** was calculated to be 0.28 min^−1^·mM^−1^. Compound **94** also showed good lipophilicity (ClogP = −0.48) [77]. A new series of substituted tetrazoles were reported where all the synthesized compounds exhibited anticonvulsant activity against subcutaneous metrazole (ScMet) and maximal electroshock (MES) induced seizures in mice. Compounds **95** and **96** proved to be the most active compounds in this study, with special high activity in the ScMet test (% protection: 100% and 80%, respectively) [78].

A series of 5-alkoxytetrazolo-[1,5-*c*]thieno[2,3-*e*]pyrimidine derivatives were synthesized and evaluated for anticonvulsant activities. The pharmacological results showed that only four of the synthesized compounds had weak anticonvulsant activity. The more active compounds were **97** and **98** [79]. A new series of 6-alkyoxytetrazolo[5,1*-a*]phthalazine derivatives was synthesized and their anticonvulsant activity evaluated. The pharmacological results showed that compound **99** was the most potential agent, with an ED_50_ value of 6.8 mg/kg and a TD_50_ value of 456.4 mg/kg. The protective index (PI = TD_50_/ED_50_) for compound **99** was 67.1, which was significantly higher than that for the reference drug carbamazepine (PI = 6.4) [80]. In our laboratory studies of antiepileptic drugs, this was found to be the best active compound in ten years. It is in preclinical stages and is expected to be eventually registered as an approved drug.

### 3.5. Antihypertensive Activity

**Figure 18:**
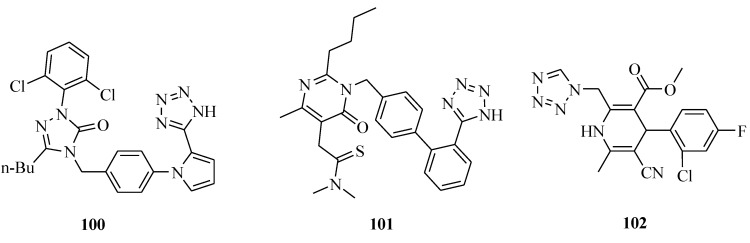
Structures of compounds **100**–**102**.

A novel nonpeptide angiotensin AT_1_ receptor antagonist was reported (Figure 18). The pharmacology results demonstrated that compound **100** inhibited [^125^I] angiotensin II binding to AT_1_ receptors in rat liver membranes (K_i_ = 2.5 ± 0.5 nM) and did not interact with AT_2_ receptors in bovine cerebellar membranes. Compound **100** inhibited the contractile response to angiotensin II (pD(2)' values: 7.43 and 7.29, respectively) with a significant reduction in the maximum. The study suggested that compound **100** may be useful for treatment of hypertension [81,82]. An angiotensin II receptor antagonist which is selectivity for the AT1 receptor subtype was also reported. The concentration of compound **101** that inhibited the binding of [^125^I] Ang II to the AT_1_ receptor from rat adrenal cortex by 50% (IC_50_) was 0.13 nM, compared with 80.0 nM for losartan. Compound **101** showed excellent inhibitory activity in the contraction of isolated rabbit thoracic aorta contrasted with other ARBs such as losartan and candesartan. The antihypertensive effect of compound **101** was also verified to be maintained for 24 h [83,84]. Arhancet *et al*., reported the structure-activity relationships of the novel series of cyanoester dihydropyridines. Compound **102** showed improved *in vitro* metabolic stability and solubility without CYP inhibition liability. On the basis of its MR potency and favorable *in vitro* pharmacokinetic profile, with its moderate clearance and good half-life, compound **102** might be a suitable candidate for *in vivo* efficacy studies [85].

### 3.6. Hypoglycemic Activity

**Figure 19:**
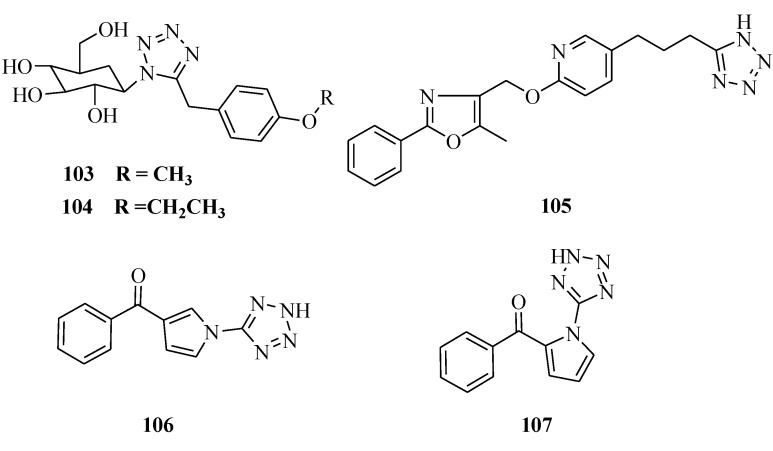
Structures of compounds **105**–**107**.

Novel tetrazole-bearing N-glycosides were designed and synthesized as SGLT2 inhibitors (Figure 19). Their hypoglycemic activity has been tested *in vivo* by a mice oral glucose tolerance test (OGTT). Two compounds were found to be more potent than the positive control dapagliflozin. The inhibition rates of blood glucose levels in mice OGTT for compounds **103** and **104** were 73.9% and 77.0% as compared with dapagliflozin (68.3%) [86]. Momose *et al.* prepared a series of 5-(4-alkoxyphenyl-alkyl)-1*H*-tetrazole derivatives and evaluated their antidiabetic effects in two genetically obese and diabetic animal models, KKA^y^ mice and Wistar fatty rats. A large number of the compounds showed potent glucose and lipid lowering activities in KKA^y^ mice. In particular, compound **105** had potent glucose lowering activity (ED_25_ = 0.0839 mg·kg^−1^·d^−1^), being 72 times more active than pioglitazone hydrochloride (ED_25_ = 6.0 mg·kg^−1^·d^−1^). This compound also showed strong glucose lowering (ED_25_ = 0.0873 mg·kg^−1^·d^−1^) and lipid lowering effects (ED_25_ = 0.0277 mg·kg^−1^·d^−1^) in Wistar fatty rats. The antidiabetic effects of the compound **105** are considered to be due to its potent agonistic activity (EC_50_ = 6.75 nM) for peroxisome proliferator-activated receptorγ (PPARγ) [87]. Pegklidou *et al*., synthesized a novel series of pyrrole based on chemotypes and evaluated their activity as selective aldose reductase inhibitors. The data indicated that the presented chemotypes **106** and **107** are promising lead compounds for the development of selective aldose reductase inhibitors, targeting the long-term complications of diabetes mellitus [88].

### 3.7. Antiparasitic Activity

**Figure 20:**
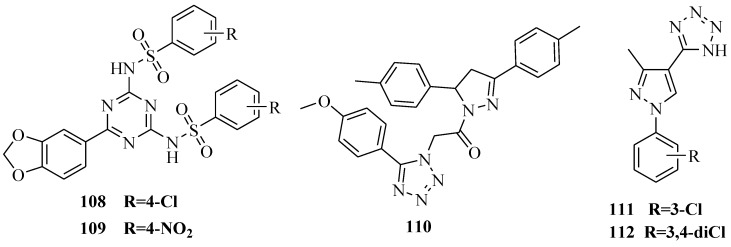
Structures of compounds **108**–**112**.

A series of compounds bearing a tetrazole and triazine ring structures conjugated with a SO_2_NH function were synthesized (Figure 20) and tested for their antiamoebic activity. Compounds **108** and **109** were the least cytotoxic (IC_50_ > 100 μM) and excellent *Entamoeba histolytica* inhibitors with IC_50_ values of 1.05 μM and 1.0 2 μM, respectively [89]. A novel series of pyrazoline derivatives were synthesized and screened *in vitro* to determine the effect on the growth of HM1:IMSS strain of *Entamoeba histolytica*. Compound **110** had potent antiamoebic activity (IC_50_= 0.86 μM), the least cytotoxicity (IC_50_ = >100 μM) and safety index value of >116.28, which was nearly twice better than metronidazole (IC_50_ > 55.55 μM) [90]. A new series of 5-(1-aryl-3-methyl-1*H*-pyrazol-4-yl)-1*H*-tetrazole derivatives and their precursor 1-aryl-3-methyl-1*H*-pyrazole-4-carbonitriles were synthesized and evaluated as *in vitro* antileishmanials against *Leishmania braziliensis* and *Leishmania amazonensis promastigotes*. In parallel, the cytotoxicity of these compounds was evaluated on the RAW 264.7 cell line. The results showed that among the assayed compounds the substituted 3-chlorophenyl (compound **111**) (IC_50_/24 h = 15 ± 0.14 μM) and 3,4-dichlorophenyl (compound **112)** (IC_50_/24 h = 26 ± 0.09 μM) tetrazoles were the most potent against *L. braziliensis* promastigotes, as compared with the reference drug pentamidine, which presented IC_50_ = 13 ± 0.04 μM. In addition, compound **111** and **112** were less cytotoxic than pentamidine [91].

### 3.8. Antiviral Activity

**Figure 21:**
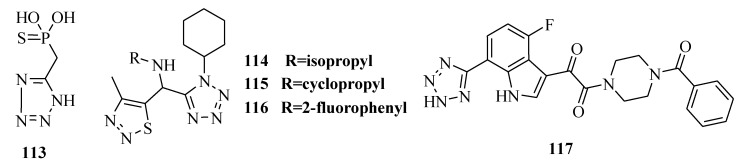
Structures of compounds **113**–**117**.

Hutchinson and Naylor prepared 5-(phosphonomethyl)-1*H*-tetrazole and a number of related tetrazoles (Figure 21) and investigated their effects on the replication of Herpes Simplex Viruses-1 and -2 as well as their abilities to inhibit the DNA polymerases induced by these viruses and the RNA transcriptase activity of influenza virus A. The thio-analogue **113** was a good chelator of zinc ions (pK_d'_ > 6, the determination of pK_d'_ values related to the strength of binding of zinc ions to phosphonates was by gel filtration) and was a more effective inhibitor of HSV-1 DNA polymerase and influenza RNA tranlscriptase [92]. A series of novel tetrazoles containing 1,2,3-thiadiazole derivatives were sythesized. The bioassay tests indicated that most of the target compounds had higher anti-TMV activity than that of ribavirin at 100 μg/mL. Compounds **114**, **115** and **116** also showed equivalent protection effect to ribavirin *in vivo* at 100 μg/mL. These studies indicated that the newly synthesized tetrazole-containing 1,2,3-thiadiazole derivatives possessed good potential bioactivities, and were worthy of further study in pesticide development [93]. 7-(2*H*-Tetrazol-5-yl)-1*H*-indole (**117**) was found to be a potent inhibitor of HIV-1 attachment but the compound was lack of oral bioavailability in rats. The cause of the low exposure was believed to be poor absorption attributed to the acidic nature of the tetrazole moiety [94,95].

### 3.9. Other Miscellaneous Activities

**Figure 22:**
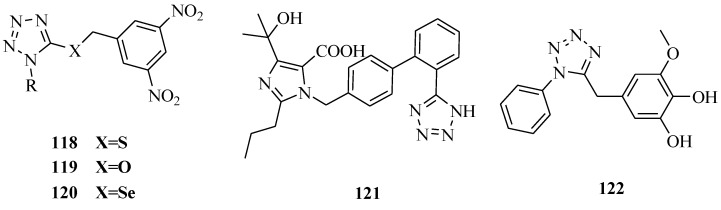
Structures of compounds **118**–**122**.

**Figure 23:**
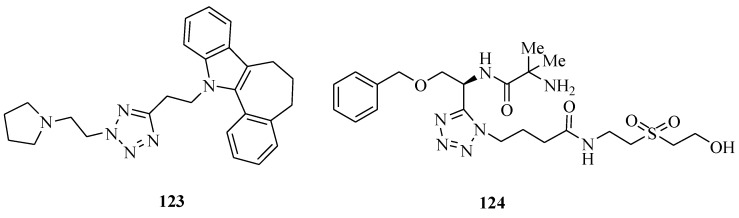
Structures of compounds **123**–**124**.

Other tetrazolium compounds that have miscellaneous activities were synthesized (Figure 22 and Figure 23). Karabanovich *et al*., reported a new class of highly potent antituberculosis agents, 1-substituted-5-[(3,5-dinitrobenzyl)sulfanyl]-1*H*-tetrazoles **118** and their oxa- and selenyl-analogs **119** and **120**. Antimycobacterial activities reached MIC values of 1 μM against *M. tuberculosis* CNCTC My 331/88 and 0.25–1 μM against six multi-drug resistant clinically isolated strains of *M. tuberculosis*; these compounds also exhibited no cross-resistance with common anti-TB drugs. Furthermore, these compounds possessed similar activity towards both INH-susceptible and INH-resistant strains of non-tuberculous *M. kansasii*. Moreover, compounds of series **118**, **119** and **120** exhibited highly selective antimycobacterial effects because they exhibit no antibacterial or antifungal effects and exhibit low toxicity on selected mammalian cell lines with IC_50_ values upwards of 30 μM [96]. Kikuchi *et al*., investigated an antiglaucoma ophthalmic agent **121** which is an angiotensin AT1 receptor antagonist with a molecular weight of approximately 446. The studies revealed that compound **121** exhibits a solubilizing effect upon insoluble additives as generally seen in detergents. Furthermore, *in vitro* permeability across rabbit corneal membrane for compound **121** decreased at higher concentrations [97]. Adibi *et al*., sythesized a series of catecholthioethers having benzoxazole and tetrazole moieties. The antioxidant activity was assessed using two methods, including 1,1-biphenyl-2-picrylhydrazyl (DPPH) radical scavenging and reducing power assays according to the methods described in the literature. All the synthesized compounds except one exhibited very good antioxidant properties. They were also more potent than BHA and Trolox used as reference compounds. The IC_50_ of compound **122** that the best for antioxidant activity among tetrazolium compounds, with a value of 0.17. Furthermore, **122** could inhibit *in vitro* growth of *Candida albicans* having the same inhibitory activity as fluconazole (MIC value 4 mg/mL) [98]. Singh *et al.* synthesized several regioisomeric tetrazolylindole derivatives and screened them for their ER binding affinity, agonist (estrogenic), antagonist (antiestrogenic) and anti-implantation activities. Compound **123** showed 100% contraceptive efficacy at 10 mg/kg dose. At a lower dose of 5 mg/kg, it was only 67% effective however [99]. Li *et al*., prepared a novel series of N1 substituted tetrazole amides and showed them to be potent growth hormone (GH) secretagogues. Among them, the most potent analog **124** (EC_50_ = 0.2 nM) showed an increase in plasma GH levels of approximately 10-fold in an anesthetized IV rat model [100].

## 4. Conclusions

All in all, some general synthetic methods, new methods of synthesis and improvements to existing procedures have been introduced that make the preparation of many tetrazole derivatives facile and efficient. This review has emphasized the diverse pharmacological properties associated with substituted tetrazoles. As evidenced by the present literature, we can find that tetrazole derivatives are a significant class of heterocyclic compounds. We firmly believe that modifications of the tetrazole moiety must help us find even more valuable biological activities. We hope that in the future more activities of tetrazole can be found and used in the field of medical sciences.
